# Evaluation of clinical efficacy of integrated traditional Chinese and Western medicine in the treatment of acute respiratory distress syndrome

**DOI:** 10.1097/MD.0000000000020341

**Published:** 2020-06-19

**Authors:** Song Zhang, Li Zhang, Kunlan Long, Peiyang Gao, Chuantao Zhang, Peng Ding, Jun Chen, Xiaoyun Zhang, Lin Qian

**Affiliations:** a Department of Critical Care Medicine; b Department of Respiratory Medicine; c Department of Emergency Medicine; d Department of Oncology, Hospital of Chengdu University of Traditional Chinese Medicine, Chengdu, China.

**Keywords:** acute respiratory distress syndrome, Clinical efficacy, integrated traditional Chinese and Western medicine, Jawei Qianyang dan, Treatment

## Abstract

**Introduction:**

Acute respiratory distress syndrome (ARDS) is a common disease in critically ill patients that has a high incidence and mortality rate worldwide. At present, there is no specific treatment for ARDS. Traditional Chinese medicine has been shown to have good potential in preventing and treating ARDS, especially in reducing the dosages of Western medicines and therefore, adverse drug reactions. The purpose of this study is to compare the clinical efficacy of integrated Chinese and Western medicine to that of Western medicine alone in the treatment of ARDS.

**Methods:**

We are proposing a prospective, multicenter, randomized, double-blind, placebo-controlled study in which 110 eligible patients would be enrolled and randomly divided into a Western medicine treatment group and an integrated Chinese and Western medicine treatment group. After 2 weeks of interventions and 1 year of follow-up, the clinical efficacy and safety of Jiawei qianyang dan in ARDS patients would be observed. The outcomes measured would include the Traditional Chinese medicine symptom score, the oxygenation index (PɑO2/FiO2), extravascular pulmonary water index, duration of mechanical ventilation, number of ICU hospitalization days, and the 28-day mortality rate for the 2 groups before and after treatment. The all-cause mortality rate, respiratory failure mortality rate, and readmission rate after 1 year of follow-up will be statistically analyzed and safety will be evaluated.

**Discussion:**

In this study, we aim to demonstrate the greater clinical efficacy of integrated traditional Chinese and Western medicine in the treatment of ARDS compared to that of Western medicine alone. In order to do this, we hope to provide evidence for the clinically supportive effect of the Jiawei qianyang dan in the treatment of ARDS and therefore demonstrate a more effective treatment.

## Abstract 2

Supplemental Digital Content is available in the text

## 1 Introduction

Acute respiratory distress syndrome (ARDS) is a common clinical disease characterized by acute hypoxemia, decreased lung compliance, and non-cardiogenic pulmonary edema, which can cause a series of serious complications and even progress to multiple organ failure.^[1–4]^ ARDS can be attributed to a variety of factors, including sepsis, multiple injuries, massive blood transfusions, pneumonia, aspiration, and lung contusions, among others. The primary pathophysiological feature of ARDS is the accumulation of a large amount of protein-rich fluid in the alveolar space due to damage of the diffuse alveolar capillary barrier.^[5–7]^ Although comprehensive studies have provided clinicians with a better understanding of the complex pathogenesis of ARDS, the incidence rate is still increasing.^[8,9]^ There is evidence that the mortality rate of ARDS patients is as high as 30% to 40% and is therefore an important cause of death in critically ill patients.^[10–15]^ Despite more than 50 years of research, there is still no specific treatment designated for ARDS, with options limited to supportive care, including protective mechanical ventilation and restriction of fluids,^[16]^ Therefore, finding more effective prevention and treatment strategies for ARDS is an urgent concern.

Traditional Chinese medicine (TCM) characteristically takes a holistic view and differentiates syndromes and treatments, potentially offering advantages and supportive effects in the management of ARDS. There is no record of a disease called ARDS in TCM. However, according to the patients’ clinical symptoms, such as shortness of breath, high fever, dry stools, crimson tongue, and the number of pulse slips, it would be diagnosed as “asthma syndrome.” The syndrome can be subdivided into “asthma syndrome” (early) and “asthma syndrome” (late). The prescriptions determined through syndrome differentiation and treatment has played a role in the diagnosis and treatment of ARDS, laying a foundation for Chinese medicine to participate in its treatment. Chinese medicine has a wide range of mechanisms to treat ARDS, mainly by regulating the cytokine network; protecting endothelial function; through antioxidation; regulating the coagulation/fibrinolysis system, immunity, and water; and through sodium transport.^[17]^

In a previous small sample study, we found that Jiawei qianyang dan (JWQYD) can reduce the extravascular lung water index and improve the prognosis of patients with ARDS, with good clinical effect.^[18]^ It is composed of aconite, Amomum, tortoise shell, ginger, licorice, and ephedra. The protective ventilation strategy adopted in the clinic for ARDS has a high risk for mechanical ventilation that can cause lung injury (VILI). In addition, VILI also releases epithelial cytokines from nonuniform lung tissue, which further aggravates lung injury and stimulates an inflammatory response.^[19,20]^ TCM can play an supportive role in ARDS treatment. Our previous study found that the JWQYD can inhibit the release of inflammatory factors in patients with ARDS; however, there is insufficient evidence to show its clinical efficacy in patients with ARDS. Therefore, a multicenter, randomized, double-blind, placebo-controlled trial should be designed to evaluate the effectiveness of JWQYD in patients with ARDS. The experimental results will provide evidence to show that JWQYD is an effective prescription for ARDS.

## 2 Method and design

### 2.1 Design

This study is a prospective, multicenter, double-blind, randomized, placebo-controlled clinical trial. The center of research participation includes 4 medical institutions in China: The Affiliated Hospital of Chengdu University of TCM, The Meishan Hospital of TCM, The Neijiang Second People's Hospital, and The ICU of Suining Hospital of TCM.

**Figure 1:**
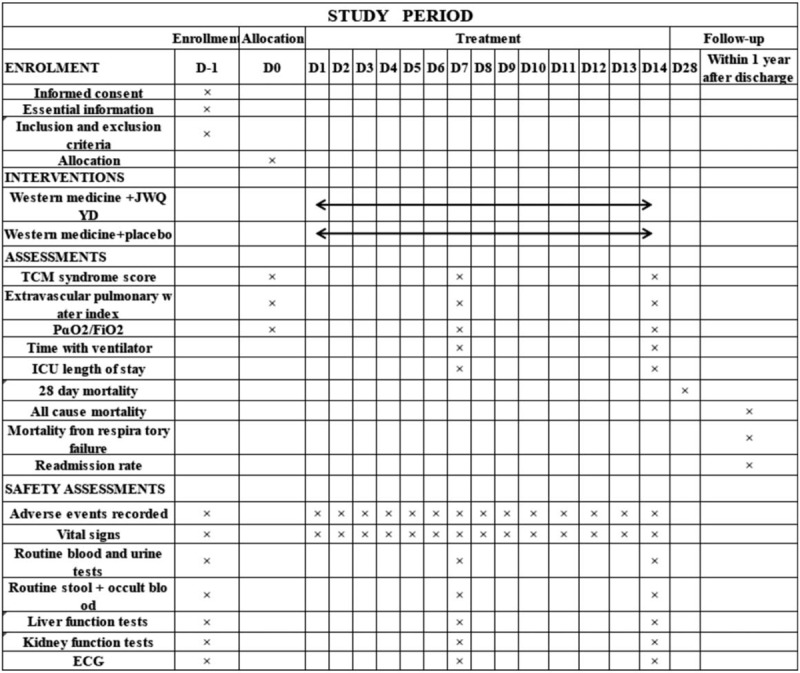
Spirit figure of enrollment, interventions, and assessments. Liver function index monitoring includes:ALP, ALT, AST, GGT, Tbil, Renal function monitoring includes:BUN, Scr, eGFR. ALP = alkaline phosphatase, ALT = alanine aminotransferase, AST = aspartate transferase, BUN = blood urea nitrogen, Scr = serum creatinine, Tbil = total bilirubin, TCM = traditional Chinese medicine, GGT = γ-glutamyl-transferase, eGFR = estimated glomerular filtration rate, PɑO2/FiO2 = oxygenation index.

**Figure 2:**
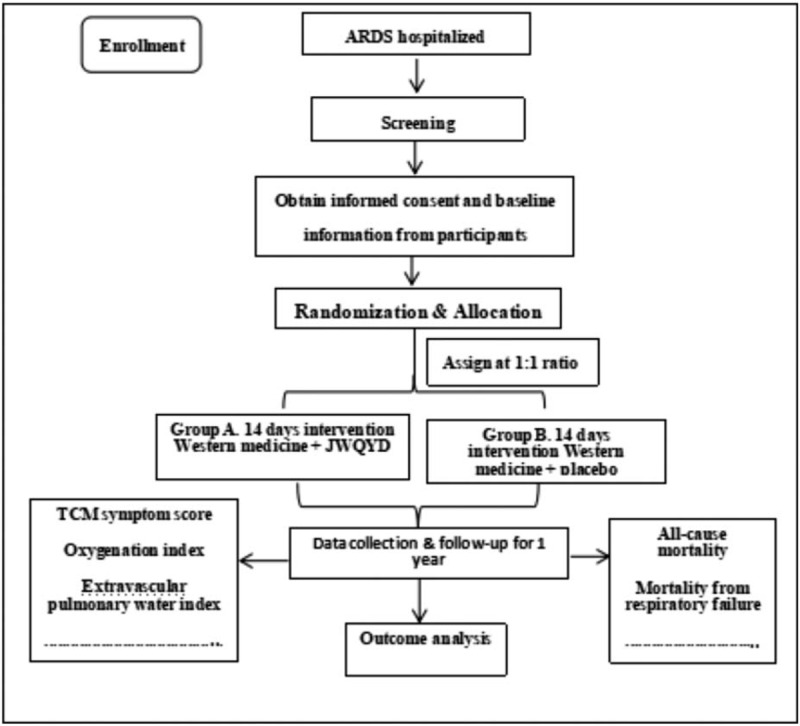
Flow chart of the study design. ARDS = acute respiratory distress syndrome, JWQYD = Jiawei qianyang dan.

The trial has been registered in the China Clinical Trial Registration Center (No. ChiCTR1900022693, which was submitted on April 23, 2019, Version.V1.4). After obtaining written informed consent, the 110 participants will be randomly divided into 2 groups at a proportion of 1:1. The 2 groups will be the integrated traditional Chinese and Western medicine treatment group and the Western medicine alone treatment group. The 2 groups of patients will receive 2 weeks of intervention treatment and 1 year of follow-up. The purpose of this experiment is to study the clinical effect of integrated traditional Chinese and Western medicine on patients with ARDS, and to evaluate the efficacy and safety of the JWQYD. At the same time, we will evaluate the long-term effect of adjuvant therapy with Chinese medicine and determine clinically effective treatments for ARDS patients. This study will follow the 2013 Standard Protocol Items: Recommendations for Interventional Trials (SPIRIT). Refer to Figure 1 for information about SPIRIT registration, intervention, and evaluation and Document 1 for the SPIRIT list.^[21]^ The study flow chart is shown in Figure 2.

### 2.2 Moral certification

This study abides by the Declaration of Helsinki (Edinburgh, 2000). The final revised draft and informed consent have been reviewed and approved by the Sichuan Regional Ethics Review Committee of The TCM/Medical Ethics Committee of The Affiliated Hospital of Chengdu University of TCM (No.2018-KL070). If any amendments are made to the agreement, the review and approval of the ethics committee will be sought again.

### 2.3 Recruitment

Participants will be recruited by doctors from the Affiliated Hospital of Chengdu University of TCM, The Meishan Hospital of TCM, The Neijiang Second People's Hospital, and the ICU of Suining Hospital of TCM. Prior to the start of the study, participants will be provided detailed information about the clinical study, including the purpose, treatment measures, schedule, and possible risks and benefits and participants or their families will be required to provide written informed consent.

### 2.4 Sample size

Calculating the sample size was based on the effective rate. According to previous research, the effective rate for the treatment group with integrated traditional Chinese and Western medicine is 65.2%, and effective rate for the group with Western medicine alone is 34.8%. The software Power Analysis and Sample Size version 11.0 (PASS 11.0)was used to calculate the sample size of the 2 groups, assuming that 10% of patients are likely to be lost during follow-up, a total of 110 patients will be enrolled.

### 2.5 Randomization and assignment blinding

Members of The Sichuan TCM Evidence-Based Medicine Center will use SAS 9.2 software (SAS, Cary, NC) to generate 110 random serial numbers. After screening and baseline evaluation, the patients with ARDS will be randomly divided into 1 of the 2 groups. Eligible patients will be assigned to the integrated traditional Chinese and Western medicine treatment group or the Western medicine alone treatment group at a proportion of 1:1. The group number will be provided in a carbon-free sealed envelope. The envelope will be kept by the study administrator, who will not be directly involved in any participant's recruitment or follow-up, and the group number will be disclosed later. The administrator will open an envelope and provide participants with their group number on the day of inclusion.

### 2.6 Blinding

The trial has a double-blind design, in which neither the researchers nor the participants will be aware of the subjects’ groups during the trial. The dosage form, color, and smell of the placebo will be similar to JWQYD. In addition, the study team will be instructed not to communicate with participants or their families about the possible grouping of the patients. Only in an emergency (such as a serious adverse event) or when the patient needs to receive emergency treatment will the investigator be required to report it to the main investigator to decide whether to expose the blind.

### 2.7 Diagnostic criteria

Participants must meet the Western medicine standard of ARDS diagnosis (Table 1),^[22]^ and the TCM syndrome judgement standard of asthma (Table 2).^[23]^ The determination of syndrome differentiation and treatment in each research center should be independently determined by 2 designated deputy chief TCM physicians.

**Table 1:**
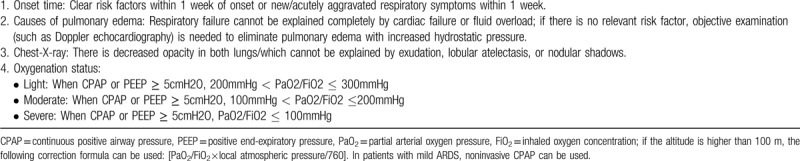
Western medicine diagnostic criteria for acute respiratory distress syndrome.

**Table 2:**
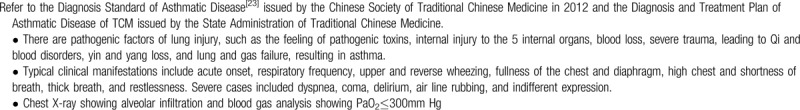
Traditional Chinese medicine criteria for syndrome of asthmatic patient.

### 2.8 Qualification criteria

#### 2.8.1 Inclusion criteria

Patients must meet all the following criteria:

(1) Aged 20 years to70 years
(2) Meet the ARDS diagnostic standard
(3) Conform to the standard of disease differentiation in TCM
(4) In order to conform to the Declaration of Helsinki and Chinese clinical trial research regulations, the patient or family members must know the content of the study and sign informed consent voluntarily.

#### 2.8.2 Exclusion criteria

Patients who meet 1 or more of the following exclusion criteria cannot be included:

(1) Pregnant or lactating women
(2) Those who are allergic to the drugs used in the program
(3) Those with surgical pointer
(4) Patients with serious bodily diseases, acquired immune deficiency syndrome, or malignant tumors
(5) Patients who may die, refuse active rescue, or are unwilling to receive Western medical treatment within 12 hours after entering the ICU
(6) Currently participating in other drug research studies
(7) Patients with severe diseases of the liver, kidney, hemopoietic system, or metabolic endocrine system, leading to organ dysfunction.

### 2.9 Termination and withdrawal criteria

All participants will be informed that they have the right to withdraw from the trial and if they do, they will also receive the standard treatment. The reason for withdrawal will be recorded in their case report file (CRF). The criteria for discontinuing treatment and withdrawing patients from the study are:

(1) The experience of adverse events related to taking the drugs, and the researchers determine that it is not appropriate to continue administering the drugs
(2) Required treatment for another serious disease during the study
(3) Poor compliance or withdrawal midway through the study
(4) Inability to tolerate 2 weeks of the intervention and cannot continue the research due to the automatic requirement of discharge or transfer
(5) Giving up treatment in advance due to economic burden and/or other factors
(6) The experience of a serious adverse event in the process of the trial, such as a life-threatening incident or even death, and the researchers could not continue

### 2.10 Test drugs

Test drugs are JWQYD decoction and JWQYD mimetic agent (placebo). The placebo will be provided by Sichuan Green Pharmaceutical Technology Development Co., Ltd. (Sichuan, China). The ingredients of JWQYD are 30 g of prepared aconite, 15 g of Amomum, 30 g of tortoise shell, 10 g of ephedra, 10 g of dried ginger, and 12 g of licorice. All the ingredients are purchased and brewed by the pharmacy department of the Affiliated Hospital of Chengdu University of TCM. The above drugs will be administered at a dose of 25 ml intranasally or orally 4 times a day (to a total of 100 mL per day). The placebo is composed of starch without any active ingredients. The color and taste of the placebo are the same as that of the TCM.

## 3 Intervention

### 3.1 Treatment plan

Both groups will be treated with Western medicine in accordance with the “Diagnosis and Treatment Guidelines for Acute Lung Injury/Acute Respiratory Distress Syndrome” formulated by the severe medicine branch of the Chinese Medical Association in 2006,^[24]^ which includes active treatment of the primary disease, respiratory support (invasive mechanical ventilation), fluid management, prostaglandin E, sedation and analgesia, and nutritional support.

Western medicine combined with JWQYD: In addition to Western medical treatment, we will administer the decoction of qianyang dan, 25 mL intranasally or orally 4 times a day.

**Western medicine:** Patients in the western medicine treatment group will receive 25 ml of the placebo intranasally or orally, 4 times a day. Index observation should be consistent with the treatment group of integrated traditional Chinese and Western medicine.

### 3.2 Determination of results

Since a holistic view of the patient is the core value of TCM, there is no single index that can predict their rehabilitation in order to evaluate the efficacy of JWQYD. Therefore, ARDS needs to be comprehensively evaluated.

1. Efficacy rate: The efficacy rate will be calculated according to the TCM syndrome score (Table 3).^[23]^ The total efficacy rate (%) = (clinical control + significant effect + effective)cases/(total cases × 100%). The score reduction (%) = (points before treatment - points after treatment)/(points before treatment × 100%). The TCM syndrome score will refer to the standard for the diagnosis and curative effect of TCM syndrome issued by the Chinese Society of TCM in 2012.

**Table 3:**
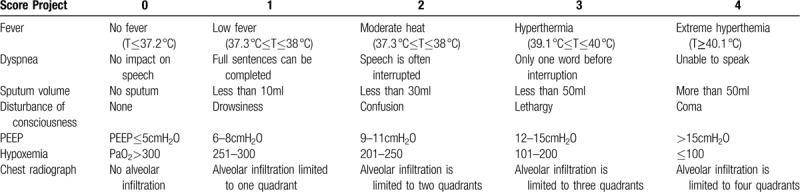
Quantitative standard for the classification of symptoms and signs.

The criteria for determining the therapeutic effect of TCM will be formulated in accordance with the guiding principles for clinical research for new TCM drugs^[25]^ (Trial) and the criteria for the therapeutic effect of the diagnosis of diseases and syndromes of TCM issued by the Chinese Society of TCM.

(1) Cure: the clinical symptoms and signs disappear or basically disappear, and the laboratory examination reveals visible improvement. The TCM syndrome score is reduced by more than 95%.
(2) Significant effect: the clinical symptoms, signs, and laboratory tests are significantly improved. The TCM syndrome score is reduced by ≥70%.
(3) Effective: the clinical symptoms, signs, and laboratory tests are improved. The TCM symptom score is reduced by ≥30%.
(4) Invalid: the clinical symptoms and signs are not improved or have worsened. There is a < 30% reduction in the TCM symptom score.

2. PɑO2/FiO2: before and after the intervention, we will perform arterial blood gas analyses to measure changes in the PɑO2/FiO2.

3. Extravascular pulmonary water index: will be obtained from the PiCCO monitor (It is a tool to detect the main hemodynamic parameters of severe patients)

4. Length of time with ventilator: the time with the ventilator will be 14 days from the patient participating in the trial to the treatment.

5. ICU length of stay: the actual length of stay = date of leaving the ICU - date of entering the ICU + 1.

6. 28-day mortality: the mortality rate from the beginning of the trial to the 28th day will be recorded.

7. Follow-up records: During the follow-up period, the all-cause mortality, respiratory failure mortality, and readmission rate in the 1-year period after discharge will be recorded. Criteria for readmission: subjects readmitted for ARDS or other respiratory diseases, not including any other disease or reason. If readmission occurs during the follow-up period, the readmission rate will not be recorded during the follow-up period. Readmission rate = number of patients readmitted/number of patients who completed follow-up.

### 3.3 Safety assessment

From baseline to the end of the study, physical examinations will be performed daily. Routine blood and urine tests, routine stool tests with occult blood, liver and renal function tests, and electrocardiograms will be performed at baseline, on day 7 and 14, and any adverse events during the study period will be observed and recorded in detail.

### 3.4 Compliance

Once the patients are randomly divided into groups, the researchers will pay close attention to the patients’ vital signs. In the case of any drug-related adverse events or other disease conditions, the physical condition of the patients will be evaluated immediately to determine whether they can continue to participate in the study and effective interventions will take place for any adverse events.

### 3.5 Adverse events

Regardless of its relationship with the study intervention, any adverse event will be recorded in the CRF. If any serious adverse event occurs, the intervention will be stopped immediately and details concerning the time, severity, relationship to the drug, and corresponding measures will be taken according to the standard operating procedures of the Chinese Food and Drug Administration. In addition, serious adverse events will be reported to the steering committee and ethics committee within 24 hours.

### 3.6 Data management and quality control

All records will be collected in the CRF and will be completed by trained and qualified investigators. After the CRF is completed, if corrections are made, the original record will not be altered, and the clinical examiner will review the completed CRF. Data input and management will be conducted under the guidance of medical statistics experts. To ensure the accuracy of the data, 2 data administrators will input and proofread the data. After checking and confirming that the established database is correct, the data will be locked by the main researchers and statistical analysts. Locked data or files will not be changed at a later date and will be submitted to the study groups for statistical analysis. The Sichuan Evidence-Based Medicine Center of TCM (Chengdu, China) has no competitive interest and will be responsible for monitoring the data. The scientific research office of the Chengdu University of TCM, which is independent of the researchers, will conduct a data review during the study.

### 3.7 Statistical analyses

Before analysis, 2 similar participants with complete data will be carefully examined to ensure that the data is correct. All data analyses will then be based on treatment intention.

The data will be analyzed using the statistical software package SPSS 22.0 (Chicago, IL). The analysis method will be selected according to the distribution characteristics of the data. The measured data will be expressed as the mean ± the standard deviation. First, the normal test and the homogeneity test of variance will be performed. In the case of normal distribution and equal variance, a *t*-test will be performed; otherwise, a nonparametric test will be utilized. The difference will be statistically significant when *P* ≤ .05.

### 3.8 Ethics and communication

The clinical trial has been reviewed and approved by the Sichuan Regional Ethics Review Committee of TCM/The Medical Ethics Committee of the Affiliated Hospital of Chengdu University of TCM (No. 2018-kl070). In order to protect the privacy of the subjects, only participating supervisors and researchers will have access to the patients’ medical records, and they will keep all patient information confidential. The ethics committee and the drug administration have the right to examine the record of clinical trials. All treatment results will be fully preserved and available to the patient and family members. The patient's research status during the follow-up period will be requested using phone interviews.

## 4 Discussion

ARDS is a major public health concern that seriously affects the quality of life and endangers the life of patients. At present, there is no specific treatment for ARDS; management primarily relies on supportive care. The economic burden of ARDS is a pressing concern worldwide.^[26,27]^ Therefore, finding effective treatment methods to improve prospects is urgent. At present, a large sample study to determine the efficacy and safety of JWQYD for the treatment of ARDS is lacking, so we will conduct a prospective randomized controlled trial to determine the risks and benefits of JWQYD in the treatment of ARDS.

This study has the following advantages: from the perspective of evidence-based medicine, this study is designed as a randomized, double-blind, placebo-controlled trial, which is considered the most authoritative research method. At the same time, this is a multicenter study conducted in 4 comprehensive Grade-A hospitals in China, which improves the external effectiveness and representativeness of the sample and reduces the risk of selection bias. Additionally, to ensure quality, all staff in the study will be required to complete a SOP (Standard Operation Procedure) training of the study procedure beforehand. Since the main stage of the study will be carried out during hospitalization, the staff will be able to quickly identify and manage adverse reactions to ensure the safety of the subjects. After discharge, researchers will maintain good telephone communication with patients and their families. However, there are some limitations to the design of the study, such as the failure to ensure that a 14-day treatment period will be the ideal length.

In summary, the purpose of this study is to determine whether JWQYD can be used as an adjuvant therapy with Western medicine in the treatment of ARDS patients, and to potentially provide objective evidence of its effectiveness and safety. If the hypothesis is confirmed, that is to say, the comprehensive plan of integrated traditional Chinese and Western medicine is relatively safe and more effective than treatment with Western medicine alone, then it can be recommended that patients with ARDS be treated with the qianyang pill to improve their clinical course.

## Acknowledgments

The authors are grateful to the Sichuan Science and Technology Program for funding this study. They also thank Editage (www.editage.cn) for English language edting.

## Author contributions

**Conceptualization:** Song Zhang, Li Zhang, Kunlan Long

**Data curation:** Lin Qian, Peng Ding, Jun Chen

**Methodology:** Li Zhang, Chuantao Zhang

**Supervision:** Peiyang Gao, Xiaoyun Zhang

**Writing – original draft:** Song Zhang, Li Zhang, Kunlan Long

**Writing-review & editing:** Peiyang Gao, Chuantao Zhang, Xiaoyun Zhang

## Supplementary Material

Supplemental Digital Content
